# Navy Department

**Published:** 1915-02

**Authors:** Joseph M. Daniels

**Affiliations:** Secretary of the Navy, Washington


### CORRESPONDENCE.

---

This department is intended for the presentation of news and views not coming within the scope of other departments, and particularly to afford opportunity for discussion and criticism of views elsewhere expressed.

---

NAVY DEPARTMENT,

Washington.

January 22, 1915.

Sir:—I am in receipt of your letter of the 14th instant, stating that you have received a complaint with regard to the stoppage of the sale of venereal prophylactic packages to enlisted men of the Navy; that the complaint seems to you to be at least in part due to interested motives; and was made to you with the request that you give the same your editorial support.

In reply, you are informed that the sale of venereal prophylactic packages aboard ship has never been authorized by the Navy Department, and could not be authorized, as the articles which may be sold in ships' stores to officers and enlisted men are prescribed by law and do not include venereal prophylactic tubes, or their equivalents, or any supplies of a medicinal nature.

The Department's attention was recently invited to certain printed circular letters advertising a prophylactic tube, the authors of which appear to be directing a systematized effort to induce enlisted men of the Navy to act as agents in the sale and distribution of the product. As such financial dealings would be in violation of Article 11, Articles for the Government of the Navy, which prescribes that "No person in the naval service shall procure stores or other articles or supplies for, and dispose thereof to, the officers or enlisted men on vessels of the Navy, or at navy yards or naval stations, for his own account or benefit," the Department brought the matter to the attention of the Commander-in-Chief, who took necessary action to circumvent the efforts made by the authors of the circular.

The foregoing perhaps explains the occasion for the complaint made to you and reported in your letter to which this is a reply.

Very respectfully,

JOSEPH M. DANIELS,

Secretary of the Navy.

Dr. A. L. Benedict,
Editor, Buffalo Medical Journal,
228 Summer Street,
Buffalo, N. Y.

